# Supplementary material for: Hexylammonium Acetate-Regulated Buried Interface for Efficient and Stable Perovskite Solar Cells
Source: Nanomaterials (Basel). 2024 Apr 9;14(8):653. doi: 10.3390/nano14080653 (PMC11055040; doi:10.3390/nano14080653)
Supplement: Supplementary file 1 [file nanomaterials-14-00653-s001.zip › nanomaterials-2955195-supplementary.pdf]

Supporting Information

Hexylammonium Acetate-Regulated Buried Interface for Efficient and Stable Perovskite Solar Cells

Experimental Section

Materials: ITO glass substrates (sheet resistance of ca. 9 Ω sq^{-1}) were purchased from OPVTECH Inc. PbI_2 (99.8%), 4-tert-butylpyridine (tBP, 96%) and bis (trifluoromethane) sulfonimide lithium salt (Li-TFSI, 99%), and ionic liquids (IL) hexylammonium acetate (HAAc) were supplied from Sigma-Aldrich. Methylammonium chloride (MACl), methylammonium bromide (MABr), and Formamidinium Iodide (FAI) were purchased from Xi'an Polymer Light Technology Crop. Spiro-OMeTAD (purity. 99.5%) was purchased from Feiming Science and Technology Co., Ltd. IPA, DMF, and DMSO were purchased from TCI. Tin (IV) oxide was purchased from Alfa Aesar. All materials were used as received without any further treatment.

Devices fabrications: ITO substrates were sequentially rinsed by sonication in detergent, deionized (DI) water and iso-propanol for 15 min, respectively, and then dried under nitrogen gas. Cleaned ITO substrates were treated with ultraviolet-ozone for 30 min, followed by deposition of a SnO_2 electron transporting layer on the substrate by spin-coating the SnO_2 QDs precursor (1.3 M) at 4000 r min^{-1} for 30 s, and subsequently annealed at 150 °C for 30 min on a hotplate. Next, HAAc was dissolved in ethanol with the concentration of 0.2, 0.5 and 1 mg/ml. The solution was spin-coated (5000 rpm, 30 s) on SnO_2 films, followed by annealing at 90 °C for 10 min. The perovskite layer was fabricated in the glovebox through a modified two-step sequential method according to the literature. First, 1.4 M PbI_2 precursor was dissolved in 950 μL DMF and 50 μL CsI solution (1.5 M, 390 mg CsI was dissolved in 1 mL DMSO). The above solution was then spin-coated on the SnO_2/ITO substrate at 1500 rpm for 30 s, and dried at 70 °C for

1 min. Thereafter, a mixture solution of FAI: MABr: MACl (90 mg: 9 mg: 9 mg in 1 mL IPA) was dropped on the PbI_2 film at 1500 rpm for 30 s. The as-cast perovskite film was annealed at 150 °C for 15 min. The spiro-OMeTAD solution was composed of 72.3 mg Spiro-OMeTAD, 30 μL TBP, and 35 μL Li-TFSI solution (260 mg in 1 mL acetonitrile) in 1 mL chlorobenzene, and then spin-coated on perovskite film at 4000 rpm for 30 s. Finally, a 100 nm Au electrode was deposited by thermal evaporation.

Characterization: The *J–V* characteristics of the PSC devices were carried out using a IVS-KA6000 Enlitech sunlight simulator equipped with an AM 1.5 filter at 100 mW cm^{-2} and Keithley SMU source after correcting the light intensity with a standard calibration cell. The corresponding EQE spectrum was acquired in air by a QE-R system from Enli Technology Co. Ltd. Static PL from 600 nm to 900 nm and TRPL measurements of perovskite film (2000 ns) were recorded by an Edinburgh FLSP1000 spectrophotometer equipped with an excitation source of 440 nm picosecond pulsed diode laser. Scanning electron microscopy (SEM) measurements of perovskite thin films were conducted to obtain the morphology on JSM-IT 800. The X-ray diffractometer spectra of the perovskite film with a scanning angle from 3° to 40° (2θ) were measured by Bruker D8 Advance. UV-vis absorption spectra of the perovskite films were obtained by a UV-3600 Plus spectrophotometer. X-ray photoelectron spectroscopy (XPS) of the perovskite films was carried out using the Thermo Kalpha XPS. ultraviolet photoelectron spectroscopy (UPS) of the perovskite films was carried out using the Thermo ESCALAB 250XIFourier-transform infrared spectra of thin films were recorded from the wavenumber 4000 to 500 cm^{-1} via Thermo Fisher Scientific Nicolet iS 50. Atomic force microscope (AFM) characterizations were conducted on an OXFORD Instruments MFP-3D Origin.

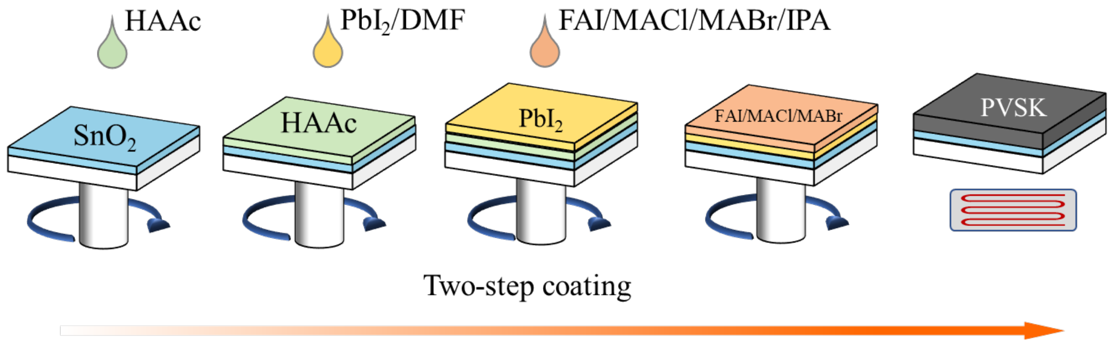

Figure S1. The preparation process using the two-step method.

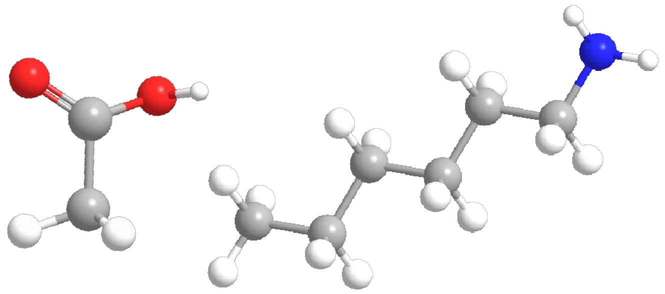

Figure S2. Chemical structures of hexylammonium acetate (HAAc). In this context, gray represents carbon atoms, white represents hydrogen atoms, red represents oxygen atoms, and blue represents nitrogen atoms.

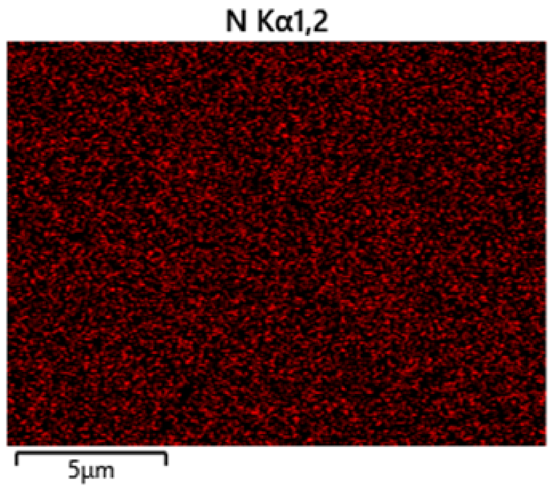

Figure S3. EDS of N for SnO_2-HAAc film.

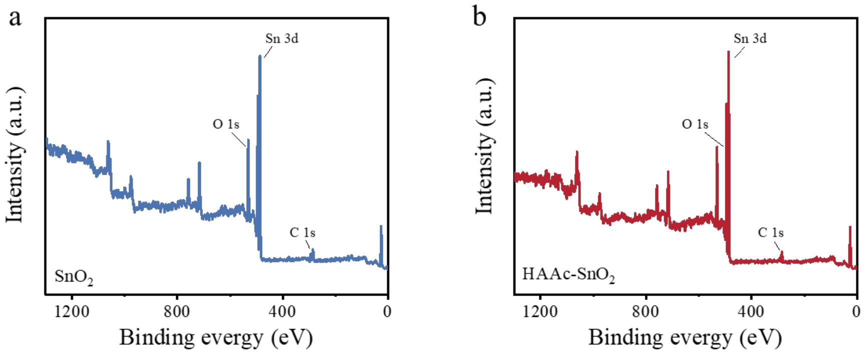

Figure S4. XPS spectra of the untreated SnO_2 and the HAAc-treated SnO_2 (HAAc-SnO_2).

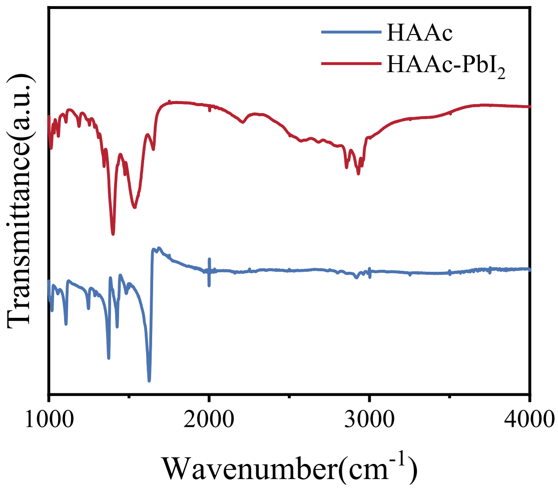

Figure S5. FTIR spectra of the IL and the IL with PbI_2 (HAAc- PbI_2).

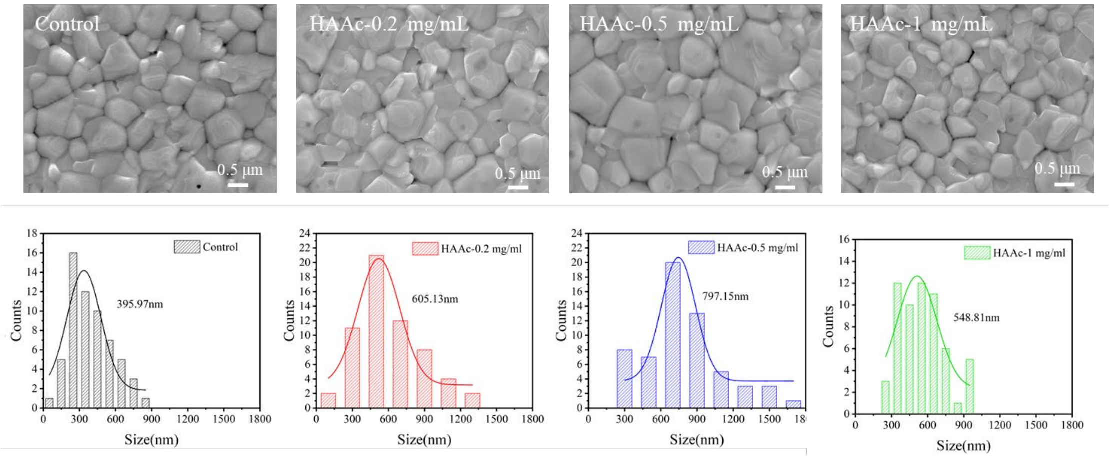

Figure S6. Top-View SEM images of perovskite film on different HAAc concentrations and the grain size distribution chart.

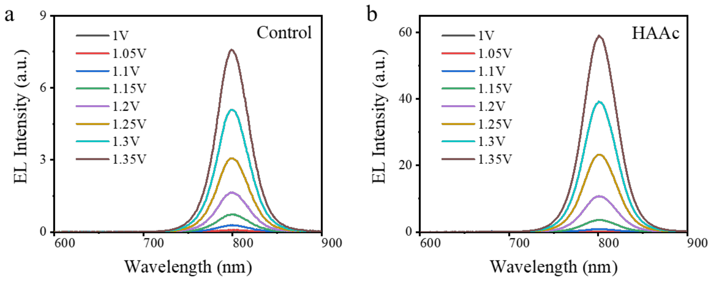

Figure S7. EQE-EL measurements for (a) control and (b) HAAc-treated PSCs under different biasing voltages.

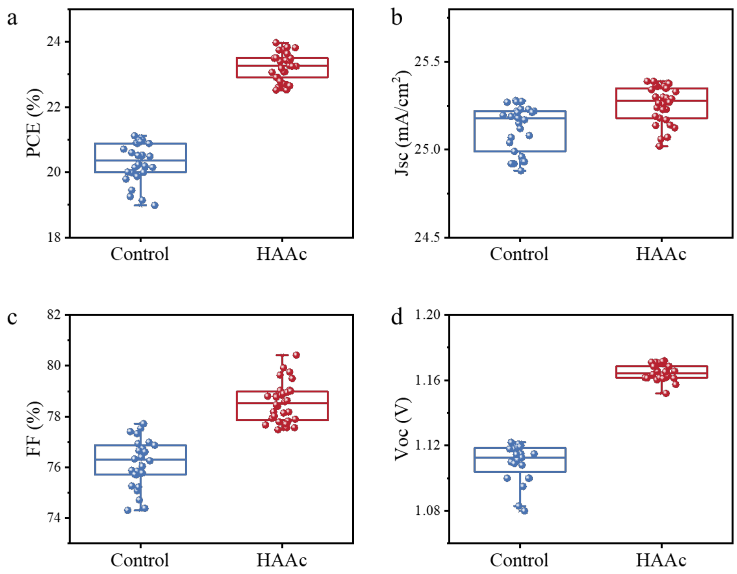

Figure S8. The statistics of photovoltaic performance parameters of HAAc-treated PSCs: PCE, *V*oc, FF, and *J*sc.

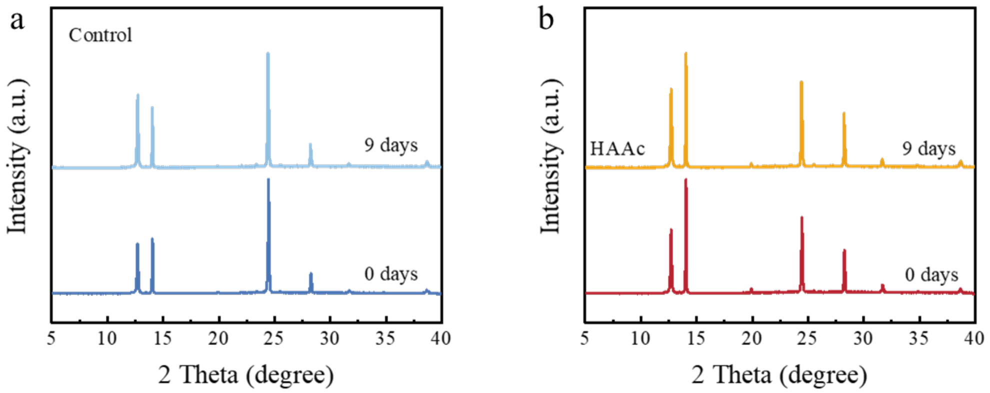

Figure S9. XRD comparison of the films after 9 days of exposure in ambient conditions at room temperature and 50% humidity.

Table S1. Detailed UPS calculation data.

Sample	Eg (eV)	$E_{on\text{-}set}$(eV)	$E_{cut\text{-}off}$ (eV)	EF (eV)	ECB (eV)	ECB (eV)
SnO_2	3.78	4.38	19.00	-2.22	-6.6	-2.82
SnO_2-HAAc	3.78	4.15	17.84	-3.38	-7.53	-3.75
ref PVSK					-5.59	-4.03

Table S2. Summary of the fitting outcomes and associated dynamic parameters extracted from TRPL decay traces.

Sample	A_1 (%)	τ_1 (ns)	A_2 (%)	τ_2 (ns)	$\tau_{avg.}$ (ns)
Control	0.39	122.55	0.36	1028.69	925.11
HAAc-modified	0.43	83.11	0.48	658.54	591.65

Table S3. The J–V curve hysteresis for the control and HAAc-modified device.

	Jsc (mA/cm^2)	Voc (V)	FF(%)	PCE(%)	HI
Control RS	25.23	1.103	75.27	20.9	0.117
Control FS	23.89	1.107	69.81	18.46	
HAAc RS	25.49	1.190	79.63	24.16	0.046
HAAc FS	24.79	1.193	77.95	23.05	
